# Machine Learning Applied to Reference Signal-Less Detection of Motion Artifacts in Photoplethysmographic Signals: A Review

**DOI:** 10.3390/s24227193

**Published:** 2024-11-09

**Authors:** Erick Javier Argüello-Prada, Javier Ferney Castillo García

**Affiliations:** 1 Programa de Bioingeniería, Facultad de Ingeniería, Universidad Santiago de Cali, Calle 5 # 62-00 Barrio Pampalinda, Santiago de Cali 760032, Colombia; 2 Programa de Mecatrónica, Facultad de Ingeniería, Universidad Autónoma de Occidente, Calle 25 # 115-85 Vía Cali-Jamundí, Santiago de Cali 760030, Colombia; jfcastillo@uao.edu.co

**Keywords:** motion artifacts, photoplethysmogram, machine learning, reference signal-less methods, real-time applications, computational complexity

## Abstract

Machine learning algorithms have brought remarkable advancements in detecting motion artifacts (MAs) from the photoplethysmogram (PPG) with no measured or synthetic reference data. However, no study has provided a synthesis of these methods, let alone an in-depth discussion to aid in deciding which one is more suitable for a specific purpose. This narrative review examines the application of machine learning techniques for the reference signal-less detection of MAs in PPG signals. We did not consider articles introducing signal filtering or decomposition algorithms without previous identification of corrupted segments. Studies on MA-detecting approaches utilizing multiple channels and additional sensors such as accelerometers were also excluded. Despite its promising results, the literature on this topic shows several limitations and inconsistencies, particularly those regarding the model development and testing process and the measures used by authors to support the method’s suitability for real-time applications. Moreover, there is a need for broader exploration and validation across different body parts and a standardized set of experiments specifically designed to test and validate MA detection approaches. It is essential to provide enough elements to enable researchers and developers to objectively assess the reliability and applicability of these methods and, therefore, obtain the most out of them.

## 1. Introduction

Out of all the sensing technologies for clinical and non-clinical applications, photoplethysmography (PPG) is arguably the one that has received the highest attention during the last decades. While initially conceived for monitoring fluctuations in peripheral blood volume, PPG technology has opened new opportunities for the non-invasive estimation of oxygen saturation [1], blood glucose levels [2], and arterial pressure [3]. PPG uses only one optical sensor, which captures light absorption changes due to the wave-like motion of the blood through the vessels, giving birth to a pulsatile waveform known as the PPG signal. However, the path light must travel through the tissues and alters during physical motion due to the relative displacement between the sensor and the skin surface, thus resulting in a severely distorted PPG waveform. As this type of disturbance can lead to erroneous estimation of several vital signs, PPG-based physiological monitoring during physical activity remains a critical challenge for the research community, which has devoted remarkable efforts to address this issue [4].

PPG signal distortions caused by body movements are commonly known as motion artifacts (MAs), and they are usually several times higher in amplitude than the signal [5]. MAs do not only significantly alter the contour of the PPG signal but also add frequency content to its power spectral density that may interfere with proper heart rate (HR) estimation based on spectral peak identification [6]. Alternatives to removing MA frequency components from the PPG spectrum include adaptive filtering and multi-resolution decomposition techniques, which have proven valuable in estimating HR accurately [7,8]. However, most MA removal and signal correction strategies are applied indistinctly to corrupted and clean segments, potentially distorting the latter and thus becoming inefficient. In this sense, there is an increasing interest in approaches that can differentiate an MA-corrupted PPG signal from a clean one.

According to Such [9], approaches for detecting MAs in biomedical signals may fall into one out of two categories: single- and multiparameter methods. Unlike single-parameter techniques, multiparameter approaches to detect MAs in PPG signals rely on additional sensors conveying information that can be associated with the motion or the signal itself. Additional sensing elements include accelerometers [10,11] and optical source-detector pairs with a peak response beyond the red–infrared wavelength range [12,13]. In some studies, the reference noise signal is generated internally from the MA-corrupted PPG segments, thus eliminating the dependency on additional hardware [14,15]. Extra sensor channels can also transmit information about the same or a similar physiological marker that responds differently to MAs. Still, using measured or synthetic reference signals to identify MA-corrupted PPG segments usually involves adaptive filtering, which, besides its high mathematical and computational complexity, may require large amounts of data and time to converge to an optimal solution [16].

Unlike the abovementioned techniques, MA detection approaches that do not require measured or synthetic reference signals (i.e., reference signal-less MA detection methods) could be more convenient for wearable, real-time applications as they overcome the need for extra data sensing and processing. Machine learning (ML) has brought remarkable advancements in this regard, as it can classify PPG signals or segments into “reliable” or “unreliable” by finding discriminatory information and identifying complex patterns, either autonomously or with minimal human intervention [17]. Several reviews on MA detection and removal from PPG signals have been published recently [5,7,8]. Nevertheless, no recent study has focused in depth on reference signal-less (RSL) methods to detect MAs in PPG signals via ML techniques. Therefore, this study aims to (i) synthesize the current state-of-the-art approaches using ML to detect MAs in PPG signals with no other information than that coming from the signal itself and (ii) provide researchers and developers with some insight to decide which method is more suitable to use for their work.

Scope: PPG signals can be distorted by factors other than motion, such as high-frequency noise, physiological processes, and environmental conditions [18,19]. Therefore, it is unfeasible to obtain an artifact-free PPG waveform in practice. In this regard, several authors have introduced the signal quality index (SQI), which aims to measure the degree of signal contamination and the reliability of the information we can extract from it [20,21,22,23]. However, given the increasing interest in developing PPG-based devices for physiological monitoring when individuals walk, exercise, or perform everyday activities, many methods for SQI estimation have been developed under the premise that motion is the factor that most distorts the PPG signal. Therefore, this review examines ML-based methods utilizing the SQI to express how contaminated the signal is mainly, or solely, due to MAs or as a feature for MA detection.

## 2. Source Identification and Selection

This narrative review was conducted by searching Scopus, IEEE Xplore, and Google Scholar using a Boolean combination of the terms “motion artifact” AND “machine learning” AND “photoplethysmogram” OR “photoplethysmography” OR “PPG.” The search was limited to conference and journal papers published in English from 2014 to 2024. Any study focusing on an ML-based approach to detect MAs in PPG signals with no measured or synthetic reference data or including it as part of a more elaborate strategy (e.g., heart rate estimation) was deemed eligible. Therefore, we excluded articles reporting techniques that filter or decompose the signal for reconstruction or quality improvement without previous identification of corrupted segments. We also discarded studies on MA-detecting approaches that require more information than that provided by the PPG signal itself. Articles outlining methodologies specifically conceived for non-contact PPG were also excluded. If several documents appeared as a sequence of incremental improvements, we selected only the one reporting the accumulation of such findings. Reviews and meta-analyses were not considered eligible, although their reference lists were used to retrieve any relevant study. Finally, we did not consider studies published as abstracts or posters.

## 3. Background on Machine Learning (ML)

Artificial intelligence (AI) encompasses all processes where machines solve problems or perform tasks mimicking human behavior [24], including a subset of techniques enabling performance improvement of computer programs from previous computations. These techniques are collectively termed machine learning (ML) and involve finding hidden insights and complex patterns without explicitly being programmed to produce the desired answer [25]. For instance, let us consider “teaching” a computer to simulate a two-input logic gate. We could use the built-in function provided by the programming environment (i.e., the explicit programming strategy) or train a classification learning algorithm (e.g., a single-layer perceptron) by presenting input data with their corresponding correct outputs according to the truth table. Thus, the computer “learns” in a supervised fashion and produces a predictive function that can deliver a binary response in the context of the given example [26]. ML techniques relying on pre-existing data labels or specifications fall under the supervised learning category. On the other hand, those algorithms detecting patterns and relationships without data specifications belong to the unsupervised learning category. Another kind of ML is reinforcement learning, in which the programmer lets the computer use the trial-and-error principle to achieve the goal by itself instead of providing input and output pairs [27]. The learner is not told which actions to take but discovers which one yields the maximum reward by trying each action iteratively.

**Figure 1:**
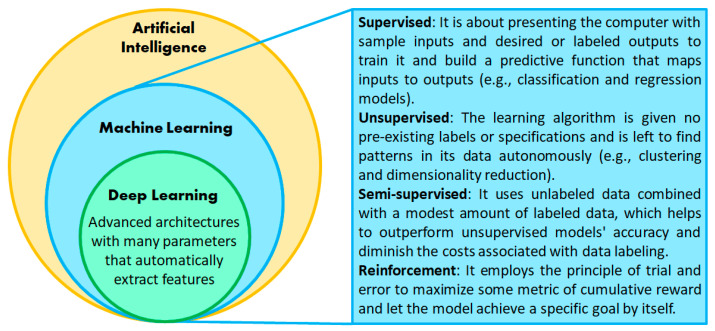
A Venn diagram-like representation of the conceptual distinction between artificial intelligence (AI), machine learning (ML), and deep learning (DL).

Deep learning is a subset of ML that does not require handcrafted feature engineering (see Figure 1) but employs a cascade of multiple layers of nonlinear processing units for automated feature extraction and transformation [28]. While layers close to data inputs identify simple features, intermediate layers use them to learn more elaborated ones. Deep learning outperforms ML when data are numerous, noisy, and unstructured. Nonetheless, responses provided by ML algorithms are more interpretable than those for deep learning algorithms [29], whose performance may decline when data dimensionality is low [30].

## 4. Machine Learning Techniques Applied to Reference Signal-Less Detection of Motion Artifacts in PPG Signals: From Traditional to Deep Learning

**Figure 2:**
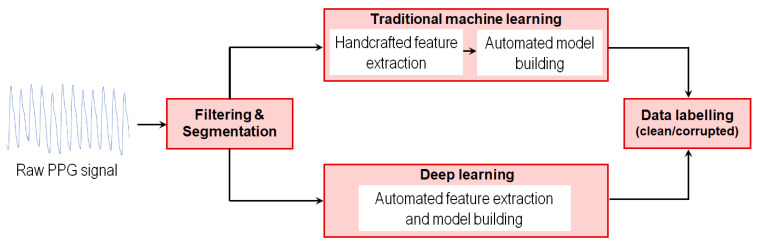
Flowchart of the processes involved in motion artifact detection from PPG signals, under the light of traditional and deep learning.

Detecting MAs in PPG signals is often addressed as a classification task in which the algorithm’s output can take only two (binary classification) or several values (multiclass classification). Traditional ML algorithms comprise classification techniques that involve manual feature extraction. On the other hand, deep learning (DL) stands for the computationally intensive processes able to automatically identify and learn discriminative feature representations with minimal or no human effort (see Figure 2). The following sections synthesize the application of traditional and deep learning techniques in the RSL detection of MA-corrupted PPG segments.

### 4.1. Traditional Machine Learning Techniques

#### 4.1.1. Characterization of Studies

Table 1 summarizes the studies using traditional ML algorithms for the RSL detection of MAs in PPG signals, with the support vector machine (SVM) being the most frequently employed classification model [31,32,33,34,35,36,37]. Only a few articles report using other classifiers, such as random forest [38,39], single-layer perceptron [40], self-organizing map [41], and elliptical envelope algorithm [42].

Most traditional ML-based approaches for MA detection in PPG signals rely on supervised learning models, and only a few studies have reported using unsupervised models. For instance, Roy and colleagues [41] employed a self-organizing map (SOM) to discriminate between clean, partially clean, and corrupted PPG segments by extracting four entropy-based features from 5 s sequences. Also known as Kohonen maps, SOMs are unsupervised learning algorithms capable of clustering groups from a high-dimensional input space onto a low-dimensional discrete lattice of output neurons [43]. In a different study, Mahmoudzadeh and co-workers [42] employed the elliptical envelope algorithm to identify MA-corrupted PPG segments from data continuously acquired over six days via a Samsung Gear Sport watch. The elliptical envelope algorithm is an unsupervised learning method that draws an ellipsoid around the center of the data samples by computing the Mahalanobis distance between them [44]. Assuming features computed over MA-free PPG segments adhere to a Gaussian distribution, data points outside the ellipsoid are deemed MA-corrupted.

#### 4.1.2. Features

The authors of the included studies have used multiple features from various domains (i.e., time, frequency, and wavelet), so there is no clear preference for a specific set of features between approaches. Time-domain features employed to detect MAs in PPG signals include those relying on fiducial point detection, such as peak-to-peak and valley-to-valley intervals, peak-to-peak and valley-to-valley amplitude differences, onset-to-peak amplitude differences, and pulse width [33,35,37,38]. Approaches using non-fiducial-based features employ statistical indexes like the mean, standard deviation, variance, interquartile range, skewness, and kurtosis of the signal [36,39,42], energy operators (e.g., Kaiser–Teager energy) [39], and entropy-based features (e.g., Shannon entropy, approximate entropy, permutation entropy, and sample entropy) [36,39,41,42]. Frequency-domain features involve the computation of the signal’s power spectral density and range from amplitudes and phases of fundamental and harmonic frequencies of the PPG signal [31,34] to spectral entropy, spectral dominant peak amplitude, and power spectral density kurtosis [32,36,39,42]. Wavelet-based features are less frequent than those from other domains, and they include measures of asymmetry, tailedness, complexity, richness, regularity, and unpredictability of approximation coefficients of the wavelet transform [36].

As important as a successful feature extraction process for traditional ML models’ performance [17] is selecting informative features with little or no redundancy. Too many features may limit the model’s performance and increase its complexity, so examining the dependency of the target variable with each computed feature and, in turn, removing the redundant ones is crucial to building more accurate and efficient models [45]. Interestingly, only three studies applying traditional ML algorithms for the RSL detection of MAs in PPG signals report feature selection methods. Two of them [39,42] used the one-way analysis of variance (ANOVA) F-test, which assesses the discriminative power of each feature by calculating the ratio of between-class variance to within-class variance [46]. The other study [31] utilized a recursive feature elimination (RFE) technique, in which an initial set of features is used to train the learning model. After computing their relevance for the classification problem, some features are eliminated, and another feature set is tested until the algorithm achieves the highest accuracy with an optimal number of features. Several other feature selection methods, such as ReliefF [47] and minimum redundancy–maximum relevance (mRMR) [48], select features based on their importance to the target variable and their redundancy with each other. However, no study applying traditional ML models to detect MA-corrupted PPG segments reports these methods.

#### 4.1.3. Benefits and Drawbacks

Traditional ML algorithms are helpful for low-dimensional data input, especially when training data availability is limited [30]. As can be seen from Table 1, the SVM is, arguably, the most widely used ML model for detecting MAs in PPG signals with no reference or extra data. SVMs can deal with nonlinear relationships between the input and output features and provide a high accuracy in exchange for a relatively short training time [26]. Just like random forest and neural networks, on the other hand, SVMs have low interpretability, which means that they do not allow an understanding of how or why they produce a specific result [49,50].

One of the main disadvantages of traditional ML algorithms is their need for a manual feature design. Handcrafted feature engineering is a time-consuming task that often requires a lot of domain expertise within an application-specific engineering process [17]. In the context of MA detection, it implies a concrete definition of how motion alters the signal morphology, which may vary depending on the movement performed by the subject. As stated before, deep learning models overcome this limitation by extracting discriminative feature representations with no or minimal human effort. They also have proven valuable in dealing with noisy, unstructured, and high-dimensional data input.

### 4.2. Deep Learning Techniques

#### 4.2.1. Characterization of Studies

Research applying deep learning techniques for the RSL detection of MAs in PPG signals is summarized in Table 2, with convolutional neural networks (CNNs) being the most widely adopted model. CNNs are the fundamental structures employed in numerous fields of ML, especially those related to computer vision and image processing [28]. Two-dimensional (2-D) image data result in a much more powerful information representation, so several authors have considered transforming the one-dimensional PPG time series into images to exploit the current advantages of CNNs. One study by Liu and colleagues [51] used the Gramian Angular Field technique [52] to encode a PPG time series into images by calculating the polar coordinates of each point of the normalized time series. The resulting matrix was used as the input of a 2-D CNN classifier with ReLU as the activation function, thus achieving an accuracy of 96.6% and 94.6% for local and publicly available datasets. Gramian Angular Fields have also been used by Suzuki and Freitas [53], who combined them with recurrence plots and Markov transition fields to obtain a hyperspectral image of 48 PPG signals extracted from twelve subjects. Several neural networks, including AlexNet, ResNet, SqueezeNet, and Swin Transformer, were tested, with most achieving higher accuracies, except for ResNet. Zargari and co-workers [54] applied a 1-D-to-2-D transformation to remove the noise induced by MAs employing a trained Cycle Generative Adversarial Network (Cycle-GAN). Nevertheless, most studies on the RSL detection of MAs report the utilization of 1-D CNNs [55,56,57,58,59,60] as it reduces the computational complexity and enables real-time classification. Shahid and colleagues [61] found that 1-D and 2-D CNNs classify time series data with a similar accuracy. Therefore, it is feasible to expect that CNNs utilizing 1-D signals directly can achieve a classification performance comparable to 2-D CNNs.

Unlike research on applying traditional ML techniques for the RSL detection of MAs, studies introducing deep learning-based approaches seem to concentrate on a narrower and more recent range of years. Possible explanations include drastically increased processing capabilities, the relative affordability of computing hardware, and recent advances in machine learning and information processing research [28,62].

**Table 2:** Summary of the deep learning-based approaches for the reference signal-less detection of motion artifacts in PPG signals.

Author(s), Year; [Reference]	Dataset	Method	Performance
Liu et al., 2020; [51]	Fifteen records selected from Physionet database + self-collected records (*n* = 15)	Two-dimensional CNN (supervised) + 10-fold CV	Physionet data Sensitivity: 94.9% Specificity: 97.8% Accuracy: 94.3% Self-collected data Sensitivity: 93.5% Specificity: 96.4% Accuracy: 96.6%
Goh et al., 2020; [55]	MIMIC II (*n* = 69) + self-collected records (*n* = 38)	One-dimensional CNN (supervised) + hold-out	Sensitivity: 96.6% Specificity: 91.2% Accuracy: 94.5%
Azar et al., 2021; [56]	Self-collected (*n* = 2)	CNN and long short-term memory (unsupervised) + hold-out	Sensitivity: 95% Precision: 90%
Guo et al., 2021; [57]	PPG-DaLiA (*n* = 15), WESAD (*n* = 15), and IEEE-SPC 2015 datasets (*n* = 12)	One-dimensional CNN with U-net architecture (supervised) + 10-fold CV	DaLiA F1-Score: 87.34 ± 0.18% WESAD F1-Score: 91.14 ± 0.33% IEEE-SPC 2015 F1-Score: 80.50 ± 1.16%
Shin, 2022; [58]	MIMIC III (*n* = 458)	One-dimensional CNN (supervised) + five-fold CV	Sensitivity: 94.8% Specificity: 99.3% Precision: 98.5% Accuracy: 97.8% F1-Score: 96.9% AUC-ROC: 98.0%
Zargari et al., 2023; [54]	Physionet-BIDMC (*n* = 53) + self-collected records (*n* = 33)	Two-dimensional Cycle Generative Adversarial Network (unsupervised) + hold-out	The peak-to-peak error and RMSE were 0.95 and 2.18 beats per minute, respectively
Freitas et al., 2023; [63]	Self-collected (*n* = 46)	Vision transformer	Sensitivity: 93.38% Precision: 94.85% Accuracy: 92.21% F1-Score: 94.11%
Lucafó et al., 2023; [59]	Self-collected (*n* = 46)	One-dimensional CNN and single-decision rule (supervised) + LOOCV	Sensitivity: 87.5 ± 0.4% Precision: 97.1 ± 0.1% Accuracy: 89.9 ± 0.2% F1-Score: 92.0 ± 0.2% AUC-ROC: 91.1 ± 0.1%
Liu et al., 2023; [64]	MIMIC III, UCI, and Queensland datasets (*n* = no reported)	Two-dimensional CNN and Swin Transformer + hold-out	Sensitivity: 97.4% Specificity: 96.1% Precision: 95.3% Accuracy: 97.3% F1-Score: 95.7% AUC-ROC: 99.2%
Suzuki and Freitas, 2024; [53]	BUTPPG dataset (*n* = 12)	SqueezeNet + hold-out	Sensitivity: 97.9% Precision: 94.4% Accuracy: 93.8% F1-Score: 95.5%
Zheng et al., 2024; [60]	Self-collected (*n* = 15)	Depth-wise separable 1-D CNN (supervised) + 10-fold CV	F1-Score: 87.20 ± 0.16%

As occurs with research on traditional ML techniques applied to the RSL detection of MA-corrupted PPG segments, unsupervised deep learning models are less frequent than their supervised counterparts. Azar and co-workers [56] proposed a CNN–long short-term memory (LSTM)-based autoencoder to detect MAs in PPG signals. The method uses the discrete wavelet transform to convert redundant samples in the signal’s temporal domain into decorrelated coefficients in the time–frequency domain, thus allowing the original samples to be compressed and depicted with fewer coefficients. Reducing the input sequences’ length enabled the model to learn the same patterns while processing fewer data points, thus achieving a sensitivity and precision of 95 and 90%, respectively. Another unsupervised model successfully adapted for MA detection was the Cycle Generative Adversarial Network (Cycle-GAN). In that study [54], 2-D representations of MA-corrupted PPG signals are detected and reconstructed by the model, which relies on several convolutional layers followed by two fully connected layers that end in a Softmax activation to compute the probability of each class (i.e., clean or noisy). The method achieved a 9.5-times improvement in MA removal compared to other non-accelerometer-based approaches.

#### 4.2.2. Automated Feature Learning

As previously pointed out, deep learning algorithms require minimal or no manual feature engineering, as they can automatically learn patterns from inputs and store them as the parameters of network connections [65]. Deep learning-based approaches eliminate the necessity to rely on parameter adjustments performed for different users, and they do not involve fiducial point detection or empirically defined features that may not hold across subjects. On the other hand, data diversity plays a significant role in how accurate deep learning-based methods can be. False classification may occur if models are trained with minimal arrhythmia-affected PPG samples [55] or lacking skin color diversity [57]. In this sense, more comprehensive datasets are necessary to produce more robust deep learning-based methods for MA detection.

## 5. Discussion

Commonly used metrics to evaluate the performance of ML-based classification algorithms include sensitivity, specificity, precision, accuracy, F1-score, and the area under the receiver operating characteristic curve (ROC-AUC). However, the metrics used to measure the effectiveness of the reviewed MA detection methods differ (see Table 1 and Table 2). Furthermore, authors used diverse datasets that include self-collected records [31,33,36,37,38,40,41,42,51,54,55,56,59,60,63], and dataset-splitting techniques are also different. This heterogeneity makes it quite difficult to systematically compare the reviewed ML-based approaches for the RSL detection of MAs, which underlines the need for using well-established reference datasets and more consistency in reporting approaches’ effectiveness. Performance differences genuinely reflecting the effectiveness of the algorithms rather than the peculiarities of the abovementioned aspects can be possible only when authors use the same datasets, data-splitting methods, and assessment metrics.

The following section will critically discuss state-of-the-art RSL methods for detecting MAs in PPG signals regarding the implications of removing or de-noising MA-corrupted PPG segments, experimental design, testing protocols, and the methods’ applicability for real-time implementations.

### 5.1. Risk of Bias in Evaluating and Reporting the Method’s Effectiveness

One crucial aspect of ML-based approaches is their ability to perform well with data different from those used for training (i.e., generalization). In this sense, it is necessary to use new, unseen data to evaluate the model’s performance; otherwise, results would be biased, depending on the model’s complexity [66]. One alternative is to divide the whole dataset into two sets: one for training the model and another for evaluation or testing. Whereas the training set allows the ML model to “learn” by updating its parameters, the test set provides a means to measure how the model reacts to new observations. In the context of the RSL detection of MA-corrupted PPG data, several strategies involving dataset splitting into training and test sets (e.g., hold-out, leave-one-out cross-validation (LOOCV), and k-fold cross-validation) have been used (see Table 1 and Table 2). However, more than half of the reviewed studies [31,32,34,37,38,40,41,42,51,53,58,59,63] do not provide clear-cut evidence indicating the utilization of unseen data (i.e., a test set) to evaluate the method’s performance. Furthermore, some articles [32,33,38,40,41,42,63] do not even report the strategy adopted to validate the proposed method or whether the training and test set distribution is the same. When authors do not take care of all these aspects, there is a risk of producing ML-based models that are extraordinarily good on paper but disappointingly average when facing unseen data [67]. It also may cast some shadows on the quality and credibility of this body of work if such aspects are not adequately outlined.

Another common pitfall of research applying ML techniques is considering single rather than multiple performance assessment metrics. Although most reviewed literature relies on several metrics when reporting the method’s performance, some studies [31,37,57,60] only considered one or two metrics (e.g., accuracy, F1-score, or both), thus hindering systematic comparisons between approaches. Accuracy can be a misleading metric for imbalanced datasets, and the F1-score, while providing a balanced view of sensitivity and precision, can be produced by multiple combinations of these two metrics [68]. Even for highly accurate ML algorithms, trade-offs are inevitable, so to help readers intuitively explore them and the implications of the model getting inaccurate, it is necessary to report not one but several well-established performance metrics. Providing information like the one displayed by confusion matrices and receiver operating characteristic (ROC) curves has also proven valuable in this context.

### 5.2. Publicly Available versus Self-Collected Records and the Experimental Design Diversity

As shown in Table 1 and Table 2, authors often employ publicly available records for benchmarking their research on MA detection methods. Data sources include the well-known Physionet’s Multiparameter Intelligent Monitoring in Intensive Care (MIMIC) dataset [69] and the subset containing physiological recordings from 53 patients admitted to medical and surgical intensive care units at the Beth Israel Deaconess Medical Center (BIDMC) [70], followed by Capnobase [71], 2015 IEEE Signal Processing Cup (SPC) Challenge [72], WESAD [73], and PPG-DaLiA [74] datasets. While publicly available datasets allow a fairer comparison among methods, PPG records from most of the abovementioned ones may not reflect disturbances produced by physical activities like walking as they come from hospitalized and ICU patients. In this sense, researchers strive to collect their data, sometimes utilizing proprietary equipment and software from manufacturers like Masimo (Masimo Corporation, Irvine, CA, USA), Sotera Digital Health (Sotera Inc., Carlsbad, CA, USA), and BIOPAC (BIOPAC Systems Inc., Goleta, CA, USA), or PPG data acquisition prototypes developed by themselves.

Concerning the strategies employed to contaminate self-collected PPG signals with MAs, some studies report that authors asked participants to wear PPG-based devices (e.g., smart watches) and perform specific actions ranging from randomly, intermittent finger or hand movements [31,33,41,54,56] to standing, walking, climbing stairs, and running on a treadmill [32,34,37,51]. However, there is no information on the duration of stationary and non-stationary conditions. In some articles, the participants could engage in daily activities while they wore the PPG acquisition device, but there were no details about the movements performed [38,42,59]. Only a few studies [34,40,51,54] report well-structured protocols (i.e., a timeline describing what actions were performed by the participants and how long they did it), which underlines the need for a standardized set of experiments specifically designed to test and validate MA detection approaches.

### 5.3. Implications of Ignoring or De-Noising MA-Corrupted Signal Segments for PPG-Based Physiological Monitoring

**Figure 3:**
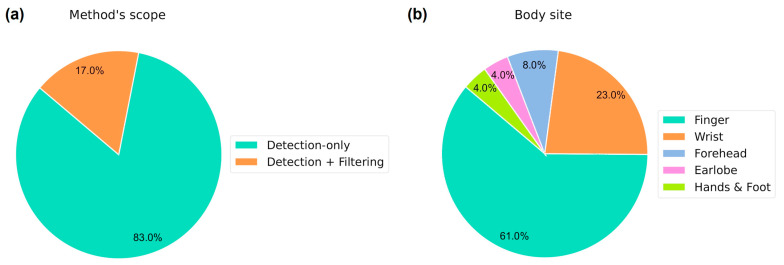
Pie charts showing (**a**) the proportion of studies according to their scope (i.e., removing versus de-noising MA-corrupted PPG segments) and (**b**) the frequency of use of several body sites across studies.

MAs may interfere with accurate PPG-based vital sign computation, so approaches able to differentiate clean from MA-corrupted signal segments may help to determine whether the value of the computed physiological index is reliable. Once the algorithm labels a PPG segment as MA-corrupted, it might be ignored or corrected using well-known or novel filtering techniques. Most of the reviewed RSL approaches have focused on assessing the usability of each signal segment [31,33,34,35,36,38,39,40,41,42,51,53,55,58,59,60,63,64], which may improve the reliability of vital sign estimation (see Figure 3a). Moreover, ignoring distorted signal segments could save computational resources and significantly reduce computing time. However, it also may lead to information loss if the subject’s movements extend for long periods or are very frequent. In this regard, RSL methods for identifying and de-noising MA-corrupted PPG segments could be more convenient for continuous PPG-based physiological monitoring. Interestingly, only a few studies fall into this subcategory [32,41,54,56].

### 5.4. Body Site Measurement

PPG-based wearable devices can operate more effectively when placed on specific body parts that do not restrict the subjects’ motion or manual activities, such as the wrist and earlobe [75]. Nevertheless, most reviewed RSL methods for MA detection rely on finger PPG data (see Figure 3b). Other body measurement sites have barely been considered, with just six [31,36,42,57,60,63], two [34,37], and one article [60] reporting the utilization of PPG data from the wrist, forehead, and earlobe, respectively. Significant differences between morphological features of finger PPG waveforms and those collected from the wrist, arm, earlobe, and forehead have been found [76]. Therefore, ML-based approaches for the RSL detection of MAs relying on finger PPG signals may not perform well in other body locations, thus limiting their applicability in the wearable industry.

### 5.5. The Promise of Real-Time Processing

Despite providing higher accuracies than rule-based methods, ML-based approaches for the RSL detection of MA-corrupted PPG signals are more computationally intensive, so they might not appear amenable to real-time implementation. Still, some attempts at online MA detection through ML techniques have been successful. Pflugradt and colleagues [40] introduced a system for Online Pulse Reliability Analysis (OPRA) using principal component analysis (PCA) and a single-layer perceptron, which is one of the most fundamental neural networks that can be implemented for solving binary classification tasks [77]. Athaya and Choi [39] used a previously trained random forest model to develop a standalone real-time application to detect MAs from the PPG signal captured by the rear camera of an Android smartphone (Google LLC, Mountain View, CA, USA). The unsupervised Cycle-GAN model proposed by Zargari and co-workers [54] takes roughly 0.4 s to detect and clean an MA-corrupted PPG signal. It also uses 45% less power than an accelerometer-based MA removal approach when implemented in a Raspberry Pi 4 (Raspberry Pi Foundation, Cambridge, UK) device for five minutes.

One critical step for implementing ML-based approaches in embedded devices is the training phase, as the computation runtime and memory consumption may increase considerably during model training. In this regard, some researchers have exploited the benefits of cloud computing to save time, memory, and power consumption by training their learning algorithms in the cloud instead of the device itself [42,60]. In another study [36], a semi-supervised one-class SVM (OCSVM) was developed for online differentiation between reliable and unreliable PPG segments. Unlike conventional SVM, OCSVM solely employs data from one class for training [78], considerably reducing computational resource usage and power consumption. All these approaches enable the utilization of the same user’s data in the training phase and real-time identification of MA-corrupted PPG segments.

While there have been remarkable efforts introducing real-time MA detection methods using ML techniques, more than half of the reviewed literature has been focused on offline processing, and no information concerning the method’s suitability for real-time implementations is provided. Several authors of RSL approaches for MA detection in PPG signals report processing time and power consumption (see Table 3). However, these measures may not be enough to claim that the method is suitable for real-time applications. One study by Faust and colleagues [79] suggests that computational complexity and speed-up are more appropriate measures to support the real-time claim. The computational complexity relates to the number of the algorithm’s executed operations as a function of the input data size [80,81], and the speed-up refers to how a parallel algorithm implementation is faster than its sequential implementation [82]. No author of any of the reviewed studies report such measures.

The computational complexity is measured using the big *O* notation and may help researchers and developers find whether a specific method is computationally suitable for real-time implementations. For instance, MA detection and removal approaches involve several signal decomposition techniques, such as principal component analysis (PCA) and empirical mode decomposition (EMD), which have been deemed computationally expensive in the past. On the other hand, Wang and co-workers [83] showed that the EMD’s complexity is equivalent to that of the fast Fourier transform (i.e., *O*(*N*log*N*), where *N* is the size of the vector to be processed). It was found that processing algorithms with a computational complexity equal to *O*(*N*log*N*) are suitable for real-time implementations [84], so it would be feasible to use EMD (and several other processing techniques) in real-time scenarios.

### 5.6. Recommendations for Future Endeavors

After identifying several challenges in the current literature on RSL methods for MA detection in PPG signals employing ML algorithms, what we consider valuable in addressing those issues is outlined below:

- Studies must provide clear-cut evidence of using new, unseen data to evaluate the proposed method and, thus, deliver an unbiased and realistic estimate of its performance. Furthermore, authors should use not one but several well-established performance metrics, as well as information like the one provided by confusion matrices and receiver operating characteristic (ROC) curves, when reporting the effectiveness of their proposed approaches;
- When relying on self-collected data, authors should include a timeline describing the actions (e.g., walking on a treadmill) performed by the participants and how long they did it. PPG recordings must contain manual annotations identifying MA-corrupted pulses or segments. In addition, all data necessary to replicate findings should be made publicly available, as suggested by an increasing number of journals and conferences;
- Given the increasing utilization of smartwatches and several other wearable devices for PPG-based physiological monitoring [75,85], researchers should design and develop their MA-detecting approaches around PPG data from body parts like the wrist, forehead, and earlobe instead of being limited to data from the fingertips;
- Authors should report objective measures, such as computational complexity and speed-up, to support the method’s suitability for real-time applications. Studies that have quantified the computational complexity of several signal decomposition and processing techniques may provide some insights into assessing the method’s suitability for identifying MA-corrupted PPG segments in real-time.

### 5.7. Limitations

A systematic comparison between studies could not be performed because their authors used different datasets, data-splitting methods, and performance assessment metrics. We did not show enough recent studies, and some potentially relevant documents might have been excluded due to a potential search engine bias (e.g., only English papers from 2014 to 2024 were included). We also did not conduct a quality assessment of the reviewed articles. Therefore, the implications and recommendations herein are limited by the lack of a standard methodological quality evaluation of the included papers.

## 6. Conclusions

Accurate identification of PPG sequences contaminated with MAs is crucial for efficient MA removal and preserving good-quality segments, and ML techniques have brought outstanding progress in the field. Nevertheless, no previous study has provided an in-depth discussion of these methods. This narrative review synthesized the current state-of-the-art approaches applying ML algorithms to detect MAs in PPG signals with no other information than that provided by the very signal. Even though there are only a few datasets where MAs are labeled in each PPG signal, supervised learning models are more frequent than their unsupervised counterparts, with SVM and CNN being the most widely used for reference signal-less-based MA detection. While traditional and deep learning-based methods show comparable performances, the latter could be more convenient for overcoming the limitations of handcrafted feature engineering. However, limitations and flaws in the current literature, particularly those regarding the model development and testing process and the measures used by authors to support the real-time claim, may prevent drawing firm conclusions about the reliability and applicability of these approaches. There is also a need for broader exploration and validation across different body parts to ensure the robustness and versatility of RSL methods for detecting MAs in PPG signals. A standardized set of experiments designed to test and validate these approaches is also necessary. Future efforts should consider providing enough proper elements to enable researchers and developers to obtain the most out of these methods.

## Figures and Tables

**Table 1:** Summary of the traditional ML-based approaches for the reference signal-less detection of motion artifacts in PPG signals.

Author(s), Year; [Reference]	Dataset	Method	Performance
Chong et al., 2014; [37]	Laboratory-controlled finger (*n* = 13), forehead (*n* = 11), and daily-activity data (*n* = 9)	SVM (supervised) + 11-fold CV	Accuracy: 94.4, 93.4, and 93.7% for laboratory-controlled finger, forehead, and daily-activity movement data, respectively. HR and SpO_2_ errors reduced to 2.3 bpm and 2.7%.
Pflugradt et al., 2015; [40]	Ten records selected from Physionet database + self-collected records (*n* = 20)	Single-layer perceptron (supervised)	Physionet data Sensitivity: 84 ± 13% Specificity: 95 ± 3% Accuracy: 89 ± 11% Self-collected data Sensitivity: 83 ± 6% Specificity: 87 ± 10% Accuracy: 82 ± 10%
Dao et al., 2016; [34]	Chon Lab (*n* = 11) + UMass Memorial Medical Center Dataset (*n* = 10)	SVM (supervised) + LOOCV	Finger Sensitivity: 92.5% Specificity: 97.5% Accuracy: 95.9% Forehead Sensitivity: 91.9% Specificity: 97.7% Accuracy: 95.5%
Karna and Kumar, 2018; [32]	IEEE-SPC 2015 (*n* = 12)	SVM (supervised)	The HR mean absolute error was 1.6 beats per minute
Sabeti et al., 2019; [35]	Capnobase (*n* = 42) + 46 records collected from acute respiratory distress syndrome databank	SVM and decision trees (supervised) + hold-out	Sensitivity: 98.27% Precision: 100.00%
Longjie and Abeysekera, 2019; [31]	Capnobase (*n* = 42) + self-collected records (*n* = 26)	SVM (supervised) + LOOCV	Accuracy: 96.6%
Subhagya and Keshavamurty, 2019; [33]	Simulated and self-collected records (*n* = no reported)	Enhanced SVM (supervised)	Sensitivity: 94.60% Specificity: 97.50% Precision: 98.57% Accuracy: 95.97%
Roy et al., 2020; [41]	Self-collected (*n* = 30)	SOM (unsupervised)	Sensitivity: 95.8% Accuracy: 92.0% F1-Score: 91.5%
Oliveira et al., 2021; [38]	Self-collected (newborns, *n* = 21)	Random forest–gradient boosting (supervised) + hierarchical rule-based approach	Sensitivity: 85.44% Specificity: 82.18% Accuracy: 84.27%
Athaya and Choi, 2021; [39]	MIMIC II (*n* = 121)	Random forest (supervised) + 10-fold CV	Sensitivity: 86.57% Specificity: 85.09% Accuracy: 85.68%
Mahmoudzadeh et al., 2021; [42]	Self-collected (women, *n* = 5)	Elliptical envelope algorithm + intra- and inter-participant CV	Sensitivity: 94.75% Precision: 94.25% F1-Score: 94.25%
Feli et al., 2023; [36]	Self-collected (*n* = 46)	SVM (supervised) + five-fold CV	Accuracy: 97.0% False Positive Rate: 1.0% AUC-ROC: 99.71%

**Table 3:** Measures provided by authors of RSL-MA detection methods based on machine learning to support the real-time claim.

Reference	Measurements	Platform
[34]	Processing time (7 ms for a 7 s PPG window length)	Intel Xeon 3.6 GHz computer
[37]	Processing time (33.3 ms for a 4 s PPG window length)	Intel Xeon 3.6 GHz computer
[40]	Processing time (181.25 ms at a sampling rate of 500 Hz) and memory usage (512 RAM bytes)	Not reported
[39]	Processing time (57.5 ms) and memory usage (15.93 KB)	Android smartphone with 4 GB RAM and 64-bit Kirin 710 processor.
[42]	Processing time (12.75 ± 0.60 ms)	Intel core i9 CPU at 2.90 GHz and 32 GB RAM
[36]	Processing time (24.71 and 26.35 ms) and power consumption (0.95 and 3.1 W)	Raspberry pi 4 and Jetson Nano
[53]	Memory usage (2 MB)	Not reported
[54]	Processing time (398 ms), memory usage (26 MB), and power consumption (3.07 W)	Raspberry pi 4
[59]	Size in disk (35.1 KB) and energy consumption (49.2 µJ per inference)	AMD EPYC 7742 64-Core Processor with 16 GB RAM
[60]	Processing time (206 ms for a 30 s PPG window length) and memory usage (134.82 RAM Bytes)	ARM 32-bit single-core Cortex-M7 processor at 216 MHz with 512 KB RAM
[64]	Processing time (515 ms for a 5 s PPG window length) and floating-point operations per second (FLOPS) (6.56)	NVIDIA RTX 3060 (12 GB VRAM) used in Python 3.9

## Data Availability

No data were generated in this work.

## Abbreviations

1-D	One-dimensional
2-D	Two-dimensional
AI	Artificial intelligence
ANOVA	Analysis of variance
CNN	Convolutional neural network
DL	Deep learning
EMD	Empirical decomposition mode
GAN	Generative Adversarial Network
HR	Heart rate
ICU	Intensive care unit
K-fold CV	K-fold cross-validation
LOOCV	Leave-one-out cross-validation
LSTM	Long short-term memory
MA	Motion artifact
ML	Machine learning
mRMR	Minimum redundancy–maximum relevance
OCSVM	One-class support vector machine
PCA	Principal component analysis
PPG	Photoplethysmogram
RFE	Recursive feature elimination
RMSE	Root mean square error
ROC	Receiver operating characteristic
RSL	Reference signal-less
SOM	Self-organizing map
SQI	Signal quality index
SpO2	Peripheral oxygen saturation
SVM	Support vector machine
